# Extracellular vesicles are transferred from melanocytes to keratinocytes after UVA irradiation

**DOI:** 10.1038/srep27890

**Published:** 2016-06-13

**Authors:** Petra Wäster, Ida Eriksson, Linda Vainikka, Inger Rosdahl, Karin Öllinger

**Affiliations:** 1 Experimental Pathology, Department of Clinical and Experimental Medicine, Linköping University, Linköping, Sweden; 2 Dermatology and Venereology, Department of Clinical and Experimental Medicine, Linköping University, Linköping, Sweden

## Abstract

Ultraviolet (UV) irradiation induces skin pigmentation, which relies on the intercellular crosstalk of melanin between melanocytes to keratinocytes. However, studying the separate effects of UVA and UVB irradiation reveals differences in cellular response. Herein, we show an immediate shedding of extracellular vesicles (EVs) from the plasma membrane when exposing human melanocytes to UVA, but not UVB. The EV-shedding is preceded by UVA-induced plasma membrane damage, which is rapidly repaired by Ca^2+^-dependent lysosomal exocytosis. Using co-cultures of melanocytes and keratinocytes, we show that EVs are preferably endocytosed by keratinocytes. Importantly, EV-formation is prevented by the inhibition of exocytosis and increased lysosomal pH but is not affected by actin and microtubule inhibitors. Melanosome transfer from melanocytes to keratinocytes is equally stimulated by UVA and UVB and depends on a functional cytoskeleton. In conclusion, we show a novel cell response after UVA irradiation, resulting in transfer of lysosome-derived EVs from melanocytes to keratinocytes.

Cells release extracellular vesicles (EVs) from at least two different origins, namely exosomes and microvesicles. Exosomes are intraluminal vesicles that are <100 nm in size and are derived from multivesicular endosomes that fuse with the plasma membrane^1^,^2^. Microvesicles, also called ectosomes, are 100 to 1,000 nm in diameter and are formed in a more rapid process than exosomes through the outward budding of the plasma membrane, which leads to the shedding of the vesicles^3^,^4^. The vesicles comprise components that are typical to their cell of origin. EVs show a preference for certain target cells, although the mechanism of recognition remains undefined^1^.

Exposure to ultra violet (UV) irradiation is the major risk factor for the development of skin cancer, in particular malignant melanoma, although the etiology of this condition involves a complex interplay between genetics, host characteristics and environmental factors^5^. The UVB component of sunlight (280–320 nm) induces DNA oxidation directly, while UVA (320–400 nm) alters the redox balance of the cell and induces oxidative stress, eventually leading to indirect DNA damage^6^,^7^. Recent studies of keratinocytes, melanocytes and melanoma cell lines have shown that UVA irradiation causes plasma membrane damage that is repaired through lysosomal exocytosis^8^,^9^,^10^.

Although lysosomes are the central degradation unit of the cell, their function goes far beyond its degradative mission and includes regulation of cell death, maintenance of cholesterol homeostasis and repair of plasma membrane damage through exocytosis^11^,^12^. Upon plasma membrane damage, Ca^2+^ influx from the extracellular compartment triggers lysosomal exocytosis and fusion with the membrane, forming a resealing patch to rescue the cell^13^,^14^,^15^. Previous studies have shown that lysosomes promote resealing through the secretion of acid sphingomyelinase (aSMase), an enzyme that generates ceramide through the cleavage of the abundant membrane lipid sphingomyelin^16^. In addition to conventional lysosomes, melanocytes harbor melanosomes, which are specialized lysosome-related organelles that contain the photoprotective pigment melanin. Melanosomes share some proteins with lysosomes but also contain unique membrane proteins, such as premelanosome protein (PMEL), tyrosinase, and tyrosinase-related protein 1, which are important for melanogenesis^17^,^18^. Furthermore, lysosomal marker proteins such as cathepsin D and lysosomal-associated membrane protein-1 (LAMP-1) are present in low levels in mature melanosomes^19^. Mature melanosomes bind microtubules and undergo bi-directional actin-dependent transport from the perinuclear area towards dendrites^20^,^21^. This transfer is stimulated and regulated through keratinocyte-derived factors^22^,^23^, although the delivery mechanism has not been fully characterized. Different mechanisms have been suggested including the heterophagocytosis of melanocyte dendrites by keratinocytes, the release of melanosome-loaded vesicles, the exocytosis of the melanin core with subsequent endocytosis by keratinocytes, transfer by nanotubes or via melanocyte filopodia, and direct fusion with the keratinocyte membrane^24^,^25^,^26^.

In previous reports, we have demonstrated the novel finding of UVA induced plasma membrane damages that is followed by repair through lysosomal exocytosis and accompanied by release of lysosomal constituents^8^,^9^. The aim of the present study was to examine UV-induced signaling between melanocytes and keratinocytes in order to determine the relationship between exocytosis of lysosomes and the transfer of melanosomes from melanocytes to keratinocytes. Interestingly, we found melanosome transfer to be mechanistically unrelated to lysosomal exocytosis. Moreover, the lysosomal exocytosis was followed by EV shedding from the melanocyte plasma membrane and uptake of EVs by keratinocytes through endocytosis.

## Results

### Lysosomal exocytosis in melanocytes is induced through UVA

**Figure 1:**
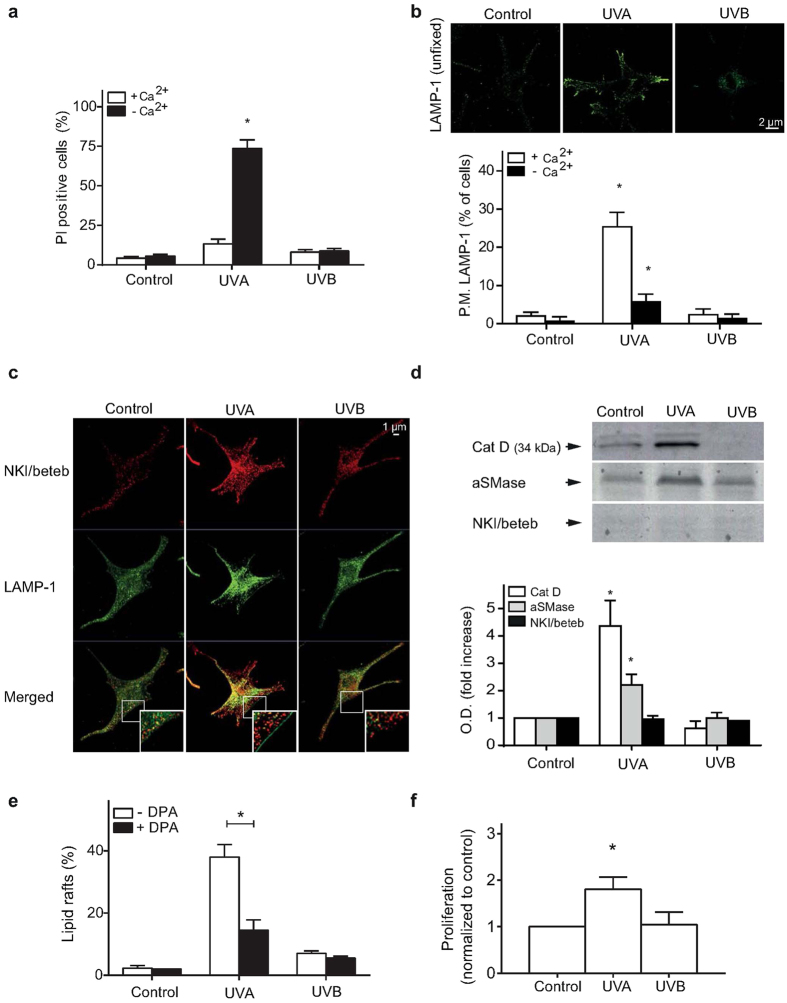
UVA irradiation induces plasma membrane damage in melanocytes, which is repaired through Ca^2+^-dependent lysosomal exocytosis. Human epidermal melanocytes in PBS with or without Ca^2+^ were exposed to UVA or UVB. (**a**) Quantification of PI positive cells, directly following irradiation in the presence or absence of Ca^2+^. PI only entered cells that failed to reseal their plasma membrane. (**b**) Immunolabeling of the luminal region of LAMP-1 in unfixed cells directly after irradiation and quantification of cells exposing LAMP-1 on the plasma membrane, and where indicated, Ca^2+^-free PBS was used. Images were selected to display the characteristic appearance of the quantified fluorescent structure. (**c**) Immunocytochemical visualization of lysosomes (LAMP-1 positive) and melanosomes (NKI/beteb positive with varying LAMP-1 positivity) in fixed melanocytes. Note only green fluorescent LAMP-1 staining at the plasma membrane in the UVA-exposed sample (inset). (**d**) Representative immunoblot of concentrated medium collected immediately after irradiation and the corresponding semiquantitative estimation of cathepsin D (Cat D), aSMase and NKI/beteb protein levels (n=3). Full-length blots are presented in Supplementary Figs S5–7. (**e**) Quantification of lipid rafts in cells with or without pretreatment with the aSMase inhibitor desipramine (DPA; 25 μM, 2 h) detected directly after UV irradiation. (**f**) Proliferation estimated in keratinocytes 24 h after supplementation of conditioned media collected immediately after irradiation of melanocytes. The experiments were performed in quadruplicates if not stated otherwise using cells from different donors. *denotes significant difference p ≤ 0.05 compared with the respective control.

Human melanocytes in monocultures were exposed to UVA or UVB with doses selected to induce comparable levels of cell damage^27^. UV exposure was performed in PBS with or without supplementation of Ca^2+^, followed by the immediate addition of propidium iodide (which enters cells with leaky plasma membranes). Lysosomal exocytosis is a Ca^2+^-dependent process and is triggered by the influx of Ca^2+^ through the wounded plasma membrane^13^. Quantification of the propidium iodide-positive cells showed a substantial increase under Ca^2+^-free conditions after UVA irradiation (Figs 1a and S1a), indicating that UVA induced plasma membrane damage. To verify lysosomal exocytosis, the translocation of LAMP-1 to the cell surface was detected through immunostaining with an antibody directed to the luminal region of LAMP-1 in unfixed cells (Fig. 1b). The number of melanocytes positive for LAMP-1 at the plasma membrane was significantly lower under Ca^2+^-free conditions, confirming that the UVA-induced plasma membrane damage is repaired through lysosomal exocytosis (Fig. 1b). However, no uptake of propidium iodide or increase in LAMP-1 surface exposure was detected after exposure to UVB (Fig. 1a,b). Next, it was established that melanosomes did not participate in the exocytosis using fixed, permeabilized cells. As presented in Fig. 1c, the control cultures contain two kinds of vesicles; lysosomes positive for LAMP-1 and melanosomes positive for the melanosomal marker NKI/beteb and showing varying intensity of LAMP-1 staining. The LAMP-1-positive staining at the plasma membrane, detected directly after UVA, did not co-localize with NKI/beteb (Fig. 1c) suggesting that only conventional lysosomes participated in exocytosis. The translocation of LAMP-1 to the plasma membrane was accompanied by the release of the lysosomal content, including cathepsin D and aSMase, into the medium directly after UVA irradiation (Fig. 1d). UVB exposure did not induce any increased release of lysosomal enzymes. Furthermore, no immediate UV-induced release of melanosomes was detected in the supernatant using the melanosomal marker NKI/beteb (Fig. 1d). Additionally, an increase in the level of lipid rafts in the plasma membrane, determined using the fluorescent cholera toxin subunit B conjugate was detected after irradiation with UVA, but not after UVB (Fig. S1b). aSMase was inhibited by pre-exposure to desipramine (Fig. S1c). Such inhibition of aSMase using desipramine diminished the number of cells showing positive lipid raft staining (Fig. 1e). Interestingly, the addition of conditioned medium from UV irradiated melanocytes to keratinocyte cultures stimulated keratinocyte proliferation after UVA, but not UVB, as detected in a WST-1 assay (Fig. 1f). Thus, irradiation with UVA caused immediate lysosomal exocytosis accompanied by release of stimulating factors apparently of lysosomal origin.

### Characterization and cellular release of EVs

**Figure 2:**
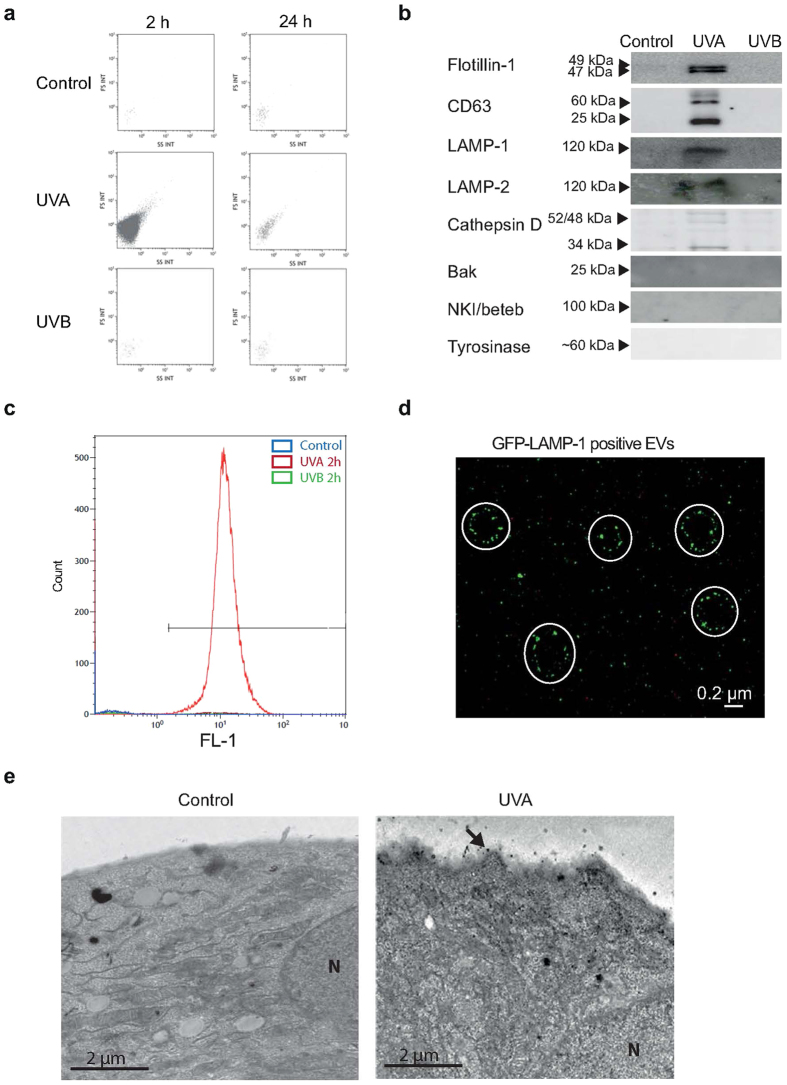
Extracellular vesicles (EVs), released from melanocytes after UVA, contain lysosomal marker proteins. Human epidermal melanocytes were irradiated with UVA or UVB. (**a**) Flow cytometric detection of microparticles released to conditioned media. (**b**) Representative immunoblots of flotillin-1, CD63, LAMP-1, LAMP-2, cathepsin D, NKI/beteb, tyrosinase and Bak in microparticles isolated from conditioned media by ultracentrifugation. Full-length blots are presented in Supplementary Figs S8–15. (**c**,**d**) Melanocytes were transfected with BacMAM GFP-LAMP-1 and EVs were isolated from conditioned media by ultracentrifugation. Analysis by (**c**) flow cytometry, and (**d**) confocal microscopy. Note vesicles with a patchy staining of LAMP-1-GFP (encircled). (**e**) EM micrographs of control and UVA exposed melanocytes. Note the irregular plasma membrane in the UVA-exposed sample. The experiments were performed in triplicates using cells from different donors. Results from one representative experiment is shown.

Conditioned media from irradiated melanocytes were collected and analyzed by flow cytometry with settings for the analysis of microparticles. Microparticles were detected in conditioned media collected 2 h after irradiation with UVA but not in controls or after UVB (Fig. 2a). Collection of conditioned media after 24 h showed reduced content of vesicles. The size and concentration of the extracellular vesicles were analyzed using an iZON QNano Nanopore instrument, which showed that UVA irradiation induced the release of vesicles that were larger than 90 nm, with a mean of 233 ± 20 nm (Table 1). Next, the vesicles were isolated by ultracentrifugation and the composition was characterized by immunoblotting. The EV markers flotillin-1 and CD63, together with the lysosomal membrane markers LAMP-1 and LAMP-2 and lysosomal cathepsin D (both pre- and catalytically active forms) were detected (Fig. 2b). However, neither the melanosome marker proteins NKI/beteb and tyrosinase, nor the mitochondrial membrane marker Bak, were found (Fig. 2b). The lysosomal origin of the EVs was verified by transfection of melanocytes with BacMAM GFP-LAMP-1 and subsequent isolation of EVs from conditioned media after UV. Using flow cytometry with setting for analysis of microparticles, a distinct peak in the FL1 channel, representing GFP-LAMP-1, was revealed after UVA (Fig. 2c) but not in controls or after UVB. Analysis of the EVs by confocal microscopy revealed vesicles visible as spheres (<0.4 μm), with patch-like LAMP-1 staining (Fig. 2d). EM micrographs presenting melanocytes after UVA revealed protrusions from the plasma membrane while the plasma membrane of controls was unaffected (Fig. 2e). These findings indicate that the EVs are budding off from the plasma membrane following lysosomal exocytosis. Interestingly, the cells were larger after UVA as found by increase in the forward scatter of flow cytometric analysis of melanocytes (Fig. S2).

### Transfer of GFP-LAMP-1 and NKI/beteb-positive vesicles to keratinocytes

**Figure 3:**
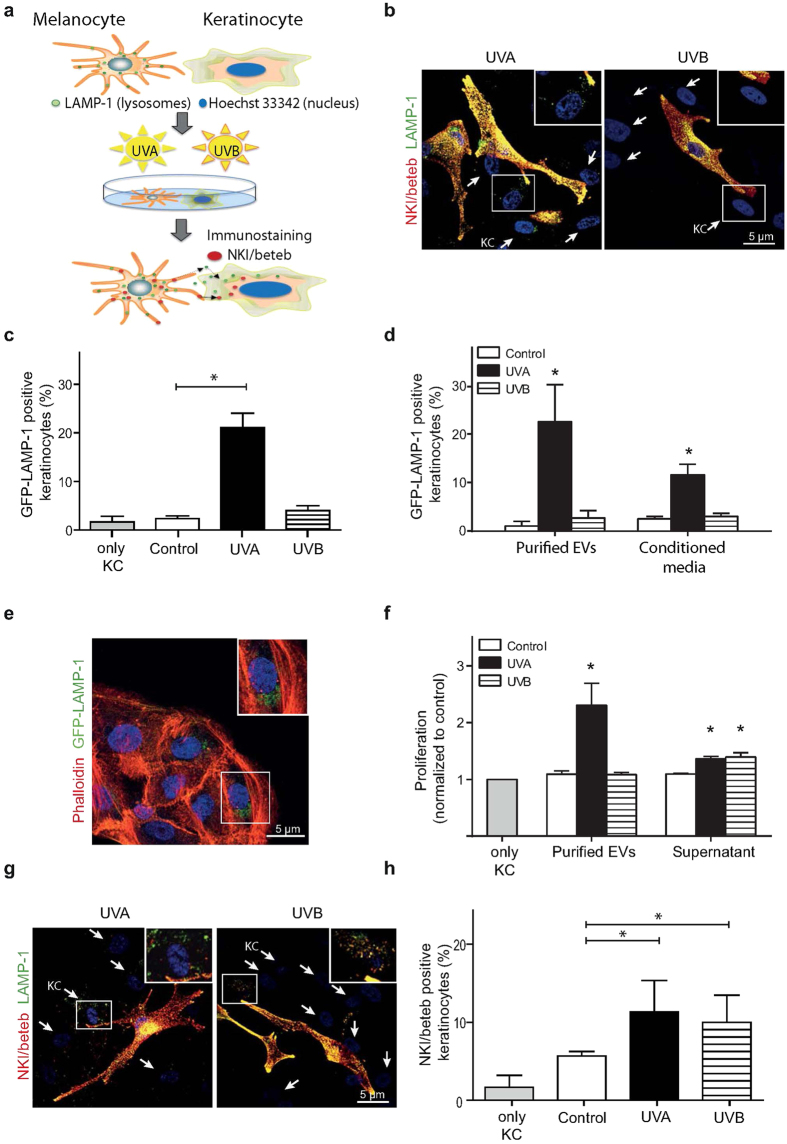
Rapid transfer of extracellular vesicles from melanocytes to keratinocytes in co-cultures after irradiation. Human epidermal melanocytes, transfected with BacMAM GFP-LAMP-1, and keratinocytes, stained with Hoechst 33342, were co-cultured for 24 h followed by irradiation with UVA or UVB. (**a**) Model of the co-culture system. (**b**) Co-cultures captured 2 h after irradiation. Note the GFP-LAMP-1 positive structures in keratinocytes (blue nuclei; arrow) and melanocytes transfected with BacMAM GFP-LAMP-1 and immunostained for NKI/beteb (melanosome marker). (**c**) Flow cytometric quantification of GFP-LAMP-1-positive keratinocytes from b. (**d**) Conditioned media was collected immediately after irradiation and either added to un-irradiated cultures or extracellular vesicles (EVs) were purified by ultracentrifugation before resuspended in medium and added to keratinocyte cultures. GFP-LAMP-1-positive cells were quantified by flow cytometry after 2 h. (**e**) Keratinocytes incubated with purified EVs for 2 h and stained with phalloidin. (**f**) Proliferation estimated in keratinocytes incubated with purified EVs or the supernatant remaining after EV isolation for 24 h. (**g**) Co-cultures captured 24 h after irradiation. Note the keratinocytes positive for GFP-LAMP and NKI/beteb. (**h**) Flow cytometric quantification of NKI/beteb-positive keratinocytes from e. The experiments were repeated 4 times using cells from different donors. *denotes significant difference p ≤ 0.05.

It is established that EVs can be transferred between cells and we hypothesized that transfer of EVs between melanocytes and keratinocytes could occur. A co-culture system of human primary melanocytes and keratinocytes from the same individual was established. To distinguish between the two cell types, melanocytes were transfected with BacMAM GFP-LAMP-1, and the nuclei of keratinocytes were stained with Hoechst 33342 prior to co-culture (Fig. 3a). Transfer of LAMP-1 positive vesicles was detected in keratinocytes 2 h after UVA using confocal microscopy (Fig. 3b). Quantification by flow cytometry of LAMP-1 positive keratinocytes (identified through Hoechst stained nuclei) confirmed that increased fluorescence was only detected after UVA and not UVB (Figs 3c and S3). Immunocytochemical staining of NKI/beteb revealed no transfer of melanosomes to keratinocytes at this same time point (Figs 3b and S4a). UVB did not induce the transfer of either GFP-LAMP-1- or NKI/beteb-positive vesicles to keratinocytes as detected after 2 h (Figs 3b,c and S4a). To further characterize the transfer of the LAMP-1-positive vesicles, we investigated whether cell-to-cell contact is required. Conditioned media from UV-irradiated melanocytes was collected directly after UV exposure and added to keratinocytes and melanocytes, respectively. Interestingly, LAMP-1 positivity was detected using flow cytometry in keratinocytes after a 2-h incubation in conditioned media from UVA irradiated cultures (Fig. 3d). In non-irradiated melanocytes incubated with conditioned medium, a small number of cells (5%) had taken up EVs (Fig. S4b). Next purified EVs from UV exposed melanocytes were added to keratinocyte cultures, which showed presence of LAMP-1 positive staining, as analyzed by flow cytometry (Fig. 3d), and in confocal microscopy where the keratinocytes were counterstained with phalloidin (Fig. 3e). A time-laps study of UVA-irradiated co-cultures confirmed the transfer of GFP-LAMP-1-containing vesicles from melanocytes to keratinocytes that were not in contact with each other (Video 1.mov). Finally, we reassessed keratinocyte proliferation by incubating cultures with purified EVs as well as the supernatant remaining after EV isolation. As presented in Fig. 3f, EVs showed a higher stimulatory effect on proliferation compared to the supernatant.

Continuing the co-culture for 24 h resulted in the significant transfer of melanosomes from melanocytes to keratinocytes, which was detected by immunostaining for NKI/beteb (Fig. 3g). Quantification of the NKI/beteb-positive keratinocytes by flow cytometry showed that UVA and UVB irradiation were equally effective in stimulating this transfer (Fig. 3h). To determine if conditioned media from irradiated cells affect tyrosinase activity, co-cultures were analyzed after supplementation of conditioned media from UVA irradiated melanocytes and keratinocytes. However, no change in tyrosinase activity was found (Fig. S4c,d).

In a previous study, we have shown that UVA also induces lysosomal exocytosis in keratinocytes^8^. Hence, we also examined whether keratinocytes produce EVs after UVA. As shown in Table 1, keratinocytes produced EVs larger than 95 nm with a mean of 180 ± 15 nm. However, after UVA irradiation, keratinocytes generated only 1% of the EVs produced by the same number of melanocytes (6.9 · 10^7^ versus 5.7 · 10^5^ particles/ml). Keratinocytes transfected with GFP-LAMP-1 and co-cultured with melanocytes showed no transfer of LAMP-1-positive vesicles (Fig. S4e). However, when conditioned medium from UVA-irradiated keratinocytes was added to un-irradiated keratinocyte cultures, a 2.5-fold increase in LAMP-1-positive vesicles was observed (Fig. S4f).

Taken together, the results from the co-culture experiments indicate that two separate types of vesicles are transferred to keratinocytes from the melanocytes at different time points after UVA exposure; vesicles positive for GFP-LAMP-1 and negative for melanosomal markers are detected in keratinocytes after 2 h, while melanosomes are detected in keratinocytes after 24 h. After UVB exposure, the transfer of GFP-LAMP-1 is negligible, whereas melanosome transfer is of the same magnitude as observed after UVA irradiation. Moreover, the EVs could be transferred without cell-to-cell contact and are preferably taken up by keratinocytes.

### Mechanism for the release of EVs and melanosomes

**Figure 4:**
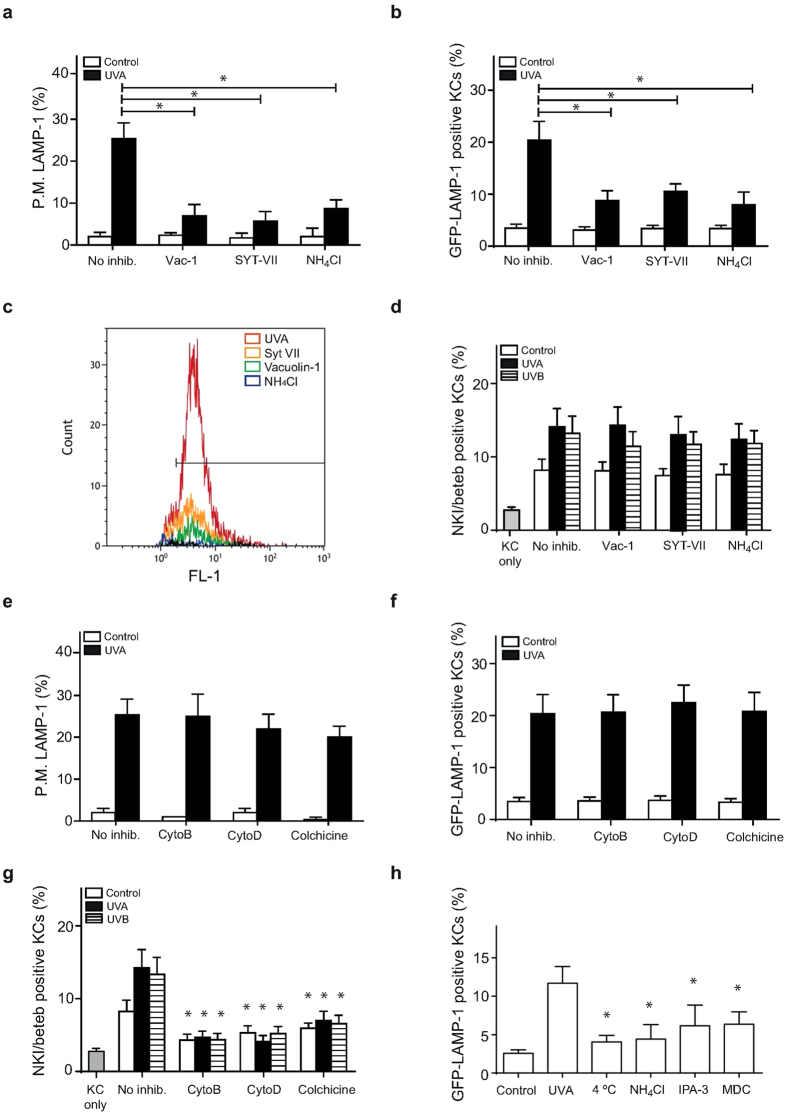
Exocytosis inhibitors prevent shedding of extracellular vesicles, while melanosome transfer is dependent on a functioning cytoskeleton. Human epidermal melanocytes **(a**,**c**,**e)** or co-cultures of melanocytes and keratinocytes **(b**,**d**,**f**,**g)** were pre-treated with vacuolin-1 (Vac-1, 1 μM for 60 min), anti-synaptotagmin VII (SYT-VII; 1 μg ml^−1^) and NH_4_Cl (10 mM, 30 min), cytochalasin B (2 μM, 60 min), cytochalasin D (2 μg ml^−1^, 60 min) or colchicine (10 μM, 30 min) as indicated, as well as irradiated with UVA or UVB. (**a**) Quantification of cells exposing the luminal part of LAMP-1 on the plasma membrane in unfixed samples directly after irradiation. (**b**) Transfer of GFP-LAMP-1 to keratinocytes analyzed 2 h after irradiation using flow cytometry. (**c**) Flow cytometric quantification of purified EVs from conditioned media isolated immediately after irradiation. (**d**) The transfer of NKI/beteb to keratinocytes analyzed 24 h after irradiation using flow cytometry. (**e**) Quantification of melanocytes exposing the luminal part of LAMP-1 in fixed samples directly after irradiation. (**f**) The transfer of GFP-LAMP-1 to keratinocytes analyzed 2 h after irradiation using flow cytometry. (**g**) The transfer of NKI/beteb to keratinocytes analyzed 24 h after irradiation using flow cytometry. (**h**) Keratinocytes were pretreated with NH_4_Cl (10 mM, 30 min), IPA-3 (2.8 nM, 30 min), MDC (200 μM, 30 min) or maintained at 4 °C prior to treatment with conditioned media collected from melanocytes immediately after UVA irradiation. GFP-LAMP-1-positive keratinocytes were analyzed after 2 h by flow cytometry. The experiments were performed in quadruplicates using cells from different donors. *denotes significant difference p ≤ 0.05 compared with irradiated sample.

No general methods to inhibit the shedding of vesicles from the plasma membrane have been identified so far. Previously, we have demonstrated that the inhibition of UVA-induced lysosomal exocytosis in keratinocytes is achieved by addition of vacuolin-1, anti-synaptotagmin VII (anti-SYTVII) and by increase in the lysosomal pH by NH_4_Cl^8^. As expected, lysosomal exocytosis was inhibited in monocultures of melanocytes after UVA exposure using these exocytosis blockers, as detected by decreased LAMP-1 exposure in unfixed melanocytes immediately after irradiation (Fig. 4a). Moreover, pretreatment with vacuolin-1, anti-SYTVII and NH_4_Cl inhibited the transfer of GFP-LAMP-1-positive EVs to keratinocytes 2 h after UVA irradiation in the co-culture system (Fig. 4b). Likewise, isolation of EVs from conditioned media and quantification by flow cytometry revealed a reduction of EVs produced in cultures exposed to the inhibitors (Fig. 4c). The effect of these inhibitors on melanosome transfer was analyzed 24 h after irradiation. However, no significant decrease in the transfer of NKI/beteb-positive vesicles was detected after irradiation with UVA or UVB (Fig. 4d). We also determined whether treatment with the actin inhibitors cytochalasin B and D and the microtubule inhibitor colchicine affected lysosomal exocytosis in monocultures of melanocytes. These results revealed no influence on lysosomal exocytosis directly after UVA exposure, which was detected through LAMP-1 staining on the plasma membrane in unfixed cells (Fig. 4e), and accordingly, the transfer of GFP-LAMP-1-positive vesicles to keratinocytes in co-cultures 2 h after UVA was not inhibited (Fig. 4f). In contrast, when analyzing the transfer of melanosomes 24 h after irradiation, a reduction of NKI/beteb-positive melanosomes was observed in cytochalasin B/D- and colchicine-treated cultures after irradiation with both UVA and UVB (Fig. 4g).

We also determined whether the uptake of EVs by the keratinocytes was affected by endocytosis inhibitors using conditioned medium from UVA-irradiated melanocytes. As shown in Fig. 4h, incubating the cultures at 4 °C prevented EV uptake. Furthermore, the neutralization of the lysosomes by exposure to NH_4_Cl, pretreatment with the macropinocytosis inhibitor IPA-3, or monodansylcadaverine (MDC), which inhibits clathrin-dependent endocytosis, also diminished the uptake of LAMP-1-positive EVs (Fig. 4h). Taken together, these results suggest that the production of EVs is associated with lysosomal exocytosis, as the same inhibitors prevent both processes, and melanosome transfer is a mechanistically unrelated process that is dependent on cytoskeletal function.

## Discussion

The results of the present study show that UVA-induced plasma membrane damage in melanocytes is rapidly repaired through Ca^2+^-dependent lysosomal exocytosis, exposing LAMP-1 on the outer leaflet of the plasma membrane. Consistent with these results, we have previously shown that irradiation with UVA, but not UVB, causes lysosomal exocytosis in keratinocytes and melanoma cells^8^,^9^. Lysosomal aSMase is translocated to the damaged plasma membrane after various forms of cellular stress, such as exposure to ionophores and pore-forming bacterial toxins^16^,^28^. aSMase catalyzes sphingomyelin hydrolysis to ceramide, which forms the signaling platforms of lipid rafts. Here, we observed an aSMase-dependent increase in lipid raft formation after UVA exposure, and interestingly, Defour *et al*., recently showed that lipid rafts are required for lysosomal patch formation during exocytosis^29^.

Following lysosomal exocytosis, plasma membrane shedding and EV formation occur. We hypothesized that the primary driving force behind the plasma membrane shedding is the increase in the area generated through lysosomal exocytosis. Previously it has been shown that expansion of the membrane area will generate an unfavorable membrane tension forming a driving force to re-establish the normal area of the membrane^30^. As melanocytes were transfected with GFP-LAMP-1, it is possible to recognize the lysosomal origin of the vesicles. LAMP-1 is also detected in multivesicular bodies but analysis of the size (>100 nm) and the rapid formation (within 5 min), clearly demonstrate that the vesicles should be considered microvesicles, also called ectosomes^1^. In a longer timeframe during co-culture, a mix of vesicles cannot be ruled out, due to the lack of established specific markers of different kinds of vesicles^31^. In addition to the lysosomal membrane proteins LAMP-1 and LAMP-2, isolated EVs from conditioned media were positive for flotillin-1 and CD63, indicating plasma membrane origin^32^. A similar shedding mechanism has been suggested for so-called enlargeosome exocytosis, which is triggered by application of a Ca^2+^ ionophore in PC12–27 cells^33^.

The mechanism of plasma membrane shedding is not completely understood, and consequently, no method to prevent EV formation has been established. However, if EV shedding is a mechanism to re-establish the normal area of the plasma membrane, then this shedding would cease when lysosomal exocytosis is prevented. Consequently, we present a novel strategy to prevent the formation of EVs through the inhibition of lysosomal exocytosis. We also show that this process is actin independent. This result is consistent with the previous finding that the depolymerization of the actin cytoskeleton using latrunculin B did not prevent the appearance of LAMP-1 on the cell surface after treatment with ionophores^34^.

We establish that EVs are released from melanocytes and preferentially taken up by keratinocytes without cell-to-cell contact. Additionally, un-irradiated melanocytes also take up UVA-produced EVs from conditioned media but to a smaller extent. Interestingly, compared with keratinocytes, melanocytes are more prone to produce EVs after UVA exposure, as 100-fold more EVs were produced from the same number of cells. However, in the epidermis, the ratio between melanocytes and keratinocytes is approximately 1:30. Thus, extrapolated to the skin *in vivo*, EVs produced from keratinocytes could also have an impact. However, the mechanism for the cell-specific uptake of EVs remains elusive^1^. EVs can enter target cells either by uptake via the endocytic pathway or by fusing with the plasma membrane and releasing the vesicle content directly into the cytoplasm^2^. Here the uptake of EVs was significantly decreased by the addition of MDC, preventing clathrin dependent endocytosis, and the macropinocytosis blocker IPA-3^35^.

In addition to the uptake of EVs, keratinocytes also received melanosomes from the melanocytes. Several different mechanisms for melanosome transfer have been presented^24^,^25^,^26^. In the present study, the conditioned media was negative for melanosome marker proteins, and no transfer was detected in keratinocytes cultured in conditioned media from irradiated melanocytes. However, we cannot rule out the release of free melanin^25^. The transfer of melanosomes was not evident until after 24 h in co-cultures, and inhibitors of lysosomal exocytosis did not affect the transfer. In contrast, melanosome transfer could be prevented through the inhibition of microtubule and actin filament polymerization. This result is consistent with the finding that melanosomes move toward the microtubule plus end to reach the plasma membrane prior to secretion^36^.

The general view is that the keratinocytes control and modulate the activity of the intracellular trafficking machinery required for efficient pigment transfer from melanocytes^22^,^23^,^25^,^37^. In a recent study, Cicero *et al*., showed that exosomes secreted by keratinocytes were detected in melanocytes after 24 h of co-culture. The exosomes transferred microRNAs that enhanced melanin synthesis^22^. However, in our experimental system the EVs were released at a much earlier time point and they did not alter tyrosinase activity, suggesting that melanin synthesis was unaffected. Instead, the melanocytic derived EVs enhanced keratinocyte proliferation. We recently found that cathepsins released extracellularly exerted mitogenic stimulation on neighboring cells^9^. The proform of cathepsin D was detected in EVs and other studies have shown that the proform of cathepsin D can stimulate proliferation^38^,^39^. Here we detect the mature form of cathepsin D in the conditioned media and the proform on purified EVs. However, purified EVs showed considerably higher stimulation of keratinocyte proliferation than the supernatant. Thus, EVs might affect epidermal homeostasis *in vivo*, by stimulating renewal of keratinocytes and promote the sun-induced thickening of epidermis.

**Figure 5:**
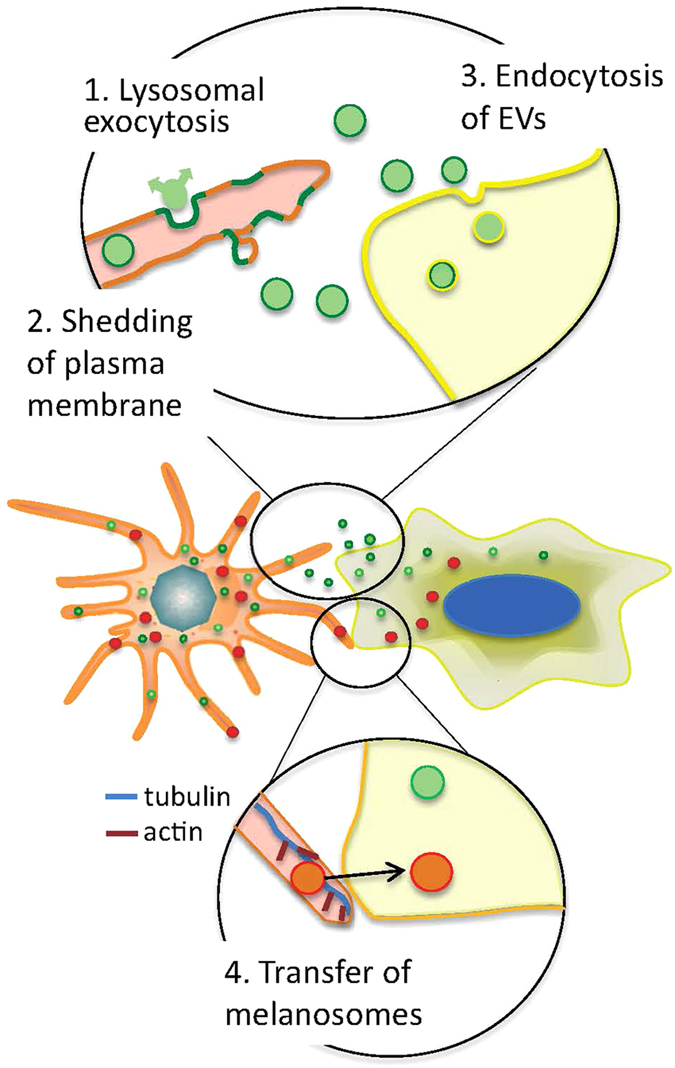
Schematic presentation of the transfer of melanosomes and EVs from melanocytes to keratinocytes. 1. Upon UVA irradiation plasma membrane damage is repaired by lysosomal exocytosis and lysosomal content is released outside the cell. 2. Plasma membrane shedding and generation of extracellular vesicles (EVs; green) by outward budding. 3. Extracellular vesicles are endocytosed by a keratinocyte. No cell-to-cell contact is needed. 4. Melanosomes (red) are transferred between melanocyte and keratinocyte in contact with each other.

In conclusion, the results of the present study provide novel insight into the mechanism underlying how UVA irradiation initiates communication between melanocytes and keratinocytes (Fig. 5). The shedding of EVs is triggered by UVA-induced plasma membrane damage and lysosomal exocytosis. While melanosomes contribute to protection against UV damage, EVs might alter proliferation rate in the recipient cells. The study discerns the melanocytes as important player in the protection against UV, not only by distribution of melanin but through rapid generation of EVs.

## Materials and Methods

### Cell cultures and conditions

All experiments were performed according to the ethical principles of the Helsinki declaration and approved by the Ethical Review Board, in Linköping, Sweden. Melanocytes, keratinocytes and fibroblasts were obtained from Caucasian donors (0–3 years of age, parental written informed consent) after foreskin circumcision, and pure cultures were established as previously described^40^. The melanocytes were cultured in Medium 199 with supplements^41^, and the keratinocytes were cultured in 3:1 Dulbecco’s minimal essential medium/Ham’s F12 with supplements^41^ at 37 °C in a humidified atmosphere with 5% CO_2_. The purity of the culture system has been confirmed via electron microscopy^42^. The experiments were conducted between passages 2–7, and no cells were cultured for more than three weeks in total. The fibroblasts were cultured in Dulbecco’s minimal essential supplemented with 10% fetal bovine serum, 1% penicillin-streptomycin and 1% streptomycin. Where indicated, the cells were transfected with Cell Light BacMAM GFP-LAMP-1 (30 particles per cell, Life Technologies Europe BV, Sweden) for 16 h prior to the experiments. For co-culture experiments, GFP-LAMP-1-transfected cells were seeded together with recipient cells, previously stained with Hoechst 33342 (2 μg/ml, 5 min, Sigma-Aldrich, St. Louis, MO, USA), at a ratio of 1:2 and incubated for 24 h. The collection of conditioned medium was performed immediately after irradiation; the medium was centrifuged at 2,000 × g for 5 min and transferred to pure cultures of the cell of interest.

Where indicated, the following supplements were added (from Sigma-Aldrich if not stated otherwise): NH_4_Cl (10 mM, pretreatment 30 min and during the experiment), anti-SYT-VII (1 μg/ml, #105172, Synaptic Systems), cytochalasin B (2 μM, 60 min), cytochalasin D (2 μg/ml, 60 min), colchicine (10 μM, 30 min), MDC (200 μM, 30 min), vacuoline-1 (1 μM, 60 min), IPA-3 (2.8 nM, 30 min), desipramine (25 μM, 2 h) or PBS without calcium supplemented with 10 mM EGTA. Corresponding solvent controls or isotype controls for antibodies supplements were analyzed in parallel, and no interference was noted.

### UV irradiation

The UVB source comprised two Philips TL20W/12 tubes (Philips, Eindhoven, the Netherlands) that emit in the spectral range of 280–370 nm and have a main output of 305–320 nm. For UVA, a Medisun 2,000-L tube (Dr Gröbel UV-Elektronik GmbH, Ettlingen, Germany; 340–400 nm) was used. A Schott WG 305 cut-off filter (50% absorption below 305 nm, Mainz, Germany) was employed, and the UVA output was 80 mW/cm^2^, while the UVB output was 1.44 mW/cm^2^. Measurements were conducted using an RM-12 (Dr Gröbel UV-Elektronik GmbH) and a PUVA Combi Light dosimeter (Leuven, Belgium). Exposure was performed in culture dishes containing pre-warmed PBS with Ca^2+^. The irradiation doses were carefully titrated to 60 J/cm^2^ for UVA and 500 mJ/cm^2^ for UVB. No increase in temperature was noted during irradiation. Following UV irradiation, fresh culture medium was added. Un-irradiated cultures were also analyzed at each time point.

### Immunocytochemistry

The cells were fixed in 4% paraformaldehyde for 20 min at 4 °C, permeabilized with 0.1% saponin and incubated 15 min with fluorescence conjugated phalloidin (0.5 μM, 15 min) or overnight at 4 °C with one of the following monoclonal anti-mouse primary antibodies: LAMP-2 (1:100, #9840-01, Southern Biotech, AL, USA), NKI/beteb (1:100, #MON7006-1, Monosan, Uden, The Netherlands); or the polyclonal anti-rabbit primary antibody tyrosinase (1:50, ab175997, Abcam, Cambridge, UK). Subsequently, the cells were incubated with the appropriate secondary antibodies conjugated to Alexa Fluor 488, Alexa Fluor 594 or Alexa Fluor 633 (1:250, Molecular Probes, Eugene, OR, USA). Next, the cells were mounted in ProLong Gold antifade reagent supplemented with 4′,6-diamidino-2-phenylindole (DAPI; Molecular Probes), and images were acquired at 20 °C using a Zeiss LSM laser scanning confocal microscope using 40X objective NA1.3 or 63X objective NA 1.4 (Carl Zeiss AG, Oberkochen, Germany). Image analysis was performed using the Zen software (Carl Zeiss AG). Negative controls, which were incubated without the primary antibody, showed no staining. Corresponding isotype controls were analyzed in parallel, and no interference was noted. To detect the luminal region of LAMP-1 at the plasma membrane, staining was performed on unfixed cells. Endocytosis was blocked by the addition of ice-cold 5% fetal bovine serum in PBS, and the samples were incubated for 5 min on ice. The cells were subsequently incubated with a primary LAMP-1 antibody (1:100, sc-8099, Santa Cruz Bio-technology; Santa Cruz, CA, USA; 2 h, 4 °C) and then fixated and incubated with a secondary antibody as described above. Quantifications were based on at least 100 cells in randomly selected areas.

### Transmission electron microscopy

Melanocyte cultures were fixed *in situ* in the plastic culture dishes by adding 2% glutaraldehyde in 0.1 M sucrose-sodium cacodylate-HCl buffer (pH 7.2) and postfixed in osmium tetroxide. Dehydration, en bloc staining with uranyl acetate, dehydration, and embedding in Epon-812 were also performed in the culture dishes. Thin sections of the cured blocks were cut with a diamond knife, stained with lead citrate, and then examined and photographed in a JEOL 2000-EX electron microscope (JEOL, Tokyo, Japan) at 100 kV.

### Plasma membrane resealing

Five minutes after UV irradiation, 5 μg ml^−1^ propidium iodide (PI; Sigma-Aldrich) was added, and the cultures were incubated for additional 5 min prior to fixation in 4% PFA (20 min, 4 °C) and mounting in ProLong Gold Antifade Reagent supplemented with DAPI (4′,6-diamidino-2-phenylindole; 1.5 μg ml^−1^; Molecular Probes). Images were captured using a fluorescence microscope (Olympus, Hamburg, Germany) and the PI positive cells were quantified by evaluation in 200 randomly selected cells per sample, n = 4.

### EV isolation

Prior to irradiation, the cells were washed in PBS with Ca^2+^ to remove any secretory vesicles. After irradiation, the conditioned media was subjected to three successive centrifugations at 2,000 × g for 10 min, 5,000 × g for 10 min and 15,000 × g for 30 min to eliminate intact cells and cellular debris. The supernatant was concentrated through ultracentrifugation for 1 h at 100,000 × g (TLA120.2 rotor, fixed-angle, titanium, Beckman Coulter Inc., USA) to pellet EVs. After isolation the EVs were resuspended in; pre-filtered exosome-free PBS for size and concentration analysis, in lysis buffer for immunoblotting and in pre-filtered exosome-free cell culture media for incubation with cells.

### Immunoblot analysis of the condition medium

Immediately after UV exposure, the conditioned medium was centrifuged at 2,000 × g for 10 min to remove cellular debris. The supernatant was concentrated using Amicon® Ultra-4 centrifugal devices (Millipore, Cork, Ireland) at 4,000 × g (40 min, 4 °C). For Western blot analysis, sample buffer (5% β-mercapto-ethanol in Laemmli sample buffer; Bio-Rad Laboratories, Hercules, CA, USA) was added, and the samples were subjected to immunoblotting as previously described^11^,^43^. Isolated EVs from 10 million cells/sample were dissolved in sample buffer (150 mM NaCl, 1% Triton X-100, 0.1% SDS, 50 mM Tris, 5 mM EDTA, pH 8.0), and the samples were loaded onto a 12% PAGE Ready gel (Bio-Rad Laboratories, Hercules, CA, USA), separated and transferred to a Hybond™-P blotting membrane (Amersham Biosciences, Buckinghamshire, UK). After saturation with 5% non-fat dry milk, immunodetection was performed using a monoclonal primary antibody against flotillin-1 (1:500, #610820, BD Biosciences, CA, USA), NKI/beteb (1:1000, #MON7006-1, Monosan), LAMP-2 (1:1000, #ab37024, Southern Biotech), CD63 (1:1000, #556019, BD Biosciences), or aSMase (1:1000, #3687, Cell Signaling Technology, MA, USA) or with a polyclonal primary antibody against cathepsin D (1:1000, #01-12-030104, Athens Research and Technology Inc., Athens, GA, USA), LAMP-1 (1:1000, sc-8099, Santa Cruz), tyrosinase (1:1000, #ab175997, Abcam) or BAK (1:1000, #12-01-16348, Biocarta). The corresponding horseradish peroxidase (mouse-, rabbit- (DAKO, Sweden), or goat- (1:2500, Santa Cruz)) conjugated secondary antibody was subsequently added. The gels have been run under the same experimental conditions and the membranes were developed using the enhanced ECL-Plus Western blotting detection system and detected on ChemiDoc MP system (Bio-Rad Laboratories). Isotype controls for respective antibodies were analyzed in parallel, and no interference was noted. Densiometric quantification of the bands was performed with Gel-Pro Analyzer 3.1 (MediaCybernetics). Full-length blots are presented in Figs S5–15.

### Detection of Lipid Rafts

The cells were incubated with the fluorescent cholera toxin subunit B conjugate (Molecular Probes) for 10 min at 4 °C, subsequently washed in PBS and cross-linked with the anti-cholera toxin B antibody for 15 min at 4 °C. The samples were processed for fluorescence microscopy and lipid rafts evaluated in 200 randomly selected cells (n = 4). Lipid rafts were visible as bright green staining at the plasma membrane and cells showing green staining of at least 10% of the surface were considered positive for lipid rafts.

### Acid-sphingomyelinase activity assay

The Amplex Red sphingomyelinase assay kit (Molecular Probes) was used to analyze aSMase activity in cells according to the manufacturer’s instructions. Fluorescence was analyzed in a VICTOR 1420 multiple counter (λ_ex_ 530–560, λ_em_590, Wallac), correlated to protein content and expressed in arbitrary units per mg protein.

### Flow cytometry

After irradiation, the cells were fixed in 4% PFA for 10 min at RT, permeabilized in 0.6% saponin and incubated with anti-mouse NKI/beteb (1:100, MON7006-1, Monosan) or anti-rabbit tyrosinase (1:100, #ab175997, Abcam) for 20 minutes. Subsequently, the cells were incubated with the secondary antibody conjugated to Alexa Fluor 633 (1:250, Molecular Probes) for 20 min at RT, rinsed with PBS, trypsinized and resuspended at a cell density of >2.5 × 10^5^ cells/mL in PBS. The samples were placed on ice and immediately analyzed using a Gallios flow cytometer (Beckman Coulter Inc., USA) equipped with the following lasers: 405-nm violet, 488-nm argon and 633-nm He/Ne. Hoechst 33342 positive cells were gated to select the recipient cell, and the fluorescence intensities of GFP-LAMP-1 and Alexa Fluor 633-conjugated NKI/beteb were analyzed in this gated population. Kaluza software (Beckman Coulter Inc.) was used to analyze the data. A total of 5,000 cells were acquired from each sample. Isotype controls and negative controls did not present any binding.

EVs were analyzed using a Gallios flow cytometer dedicated to detect submicrometer particles (discrimination set to 1). Liposomes (diameter of 50–100 nm) comprising dioleoyl phosphatidylcholine and dioleoyl phosphoethanolamine at the ratio 60:40 stained with green, and red fluorescent probes were used as positive control. From each sample, 5,000 events were acquired or events were collected during a maximum period of 5 min. Data analysis was performed using Kaluza software (Beckman Coulter Inc.).

### Size and concentration of EVs

EVs (without GFP-LAMP-1 pretreatment in order to avoid interference) dissolved in prefiltered exosome-free PBS were analyzed using an iZON QNano Nanopore instrument (calibrated using reference particles; 70, 100, 200 and 400 nm, iZON Science, Oxford, United Kingdom) with a NP100, NP150 and NP200 nanopore and the following settings: stretch 47 mm, 9 mbar, and 0.38 V. A minimum of 500 particles or a 2-minute detection was used, and the data were processed using iZON Control Suite V3.0 software, which detects the size and concentration of the vesicles.

### Live cell

Melanocytes were transfected with Cell Light BacMAM GFP-LAMP-1 and co-cultured with keratinocytes at the ratio 1:2 in 96-well plates and incubated for 24 h. After UVA irradiation, the cultures were observed using Incucyte ZOOM^®^ software (Essen Biosciences, Michigan, USA), and time-lapse phase contrast and fluorescent images were combined. Frames were captured every 10 minutes for 2 h.

### Proliferation assay

Primary cultures of melanocytes were allowed to proliferate for 24 h at 37 °C. The WST-1 cell proliferation reagent (1:10, Roche Diagnostics GmbH, Mannheim, Germany) was added and incubated for 3 hours at 37 °C. Absorbance was measured at 450 and 650 nm to determine proliferation and background absorbance, respectively, using a VICTOR X3 multiple counter (Perkin Elmer).

### Tyrosinase activity

Tyrosinase activity was estimated by analyzing the rate of oxidation of L-tyrosinse (Sigma-Aldrich). Cells from a subconfluent monolayer were suspended and mixed in 100 μl of 50 mM phosphate buffer, pH 6.8, containing 1% (w/v) TritonX-100. The extracts were cleared by centrifugation at 10,000 g for 5 min and 90 μl of each extract were added to a 96-well plate. L-Tyrosine (0.1 mM, in phosphate buffer) was added and the enzymatic reaction was followed at 550 nm for at least 1 h at 37 °C (VICTOR X3 multiple counter). The tyrosinase activity was correlated to the total amount of protein.

### Statistics

All experiments were repeated at least three times using cells from different donors, and the results are presented as the means and the standard deviations of independent samples. Statistical evaluation was performed via one-way or two-way ANOVA, followed by Dunnett´s or Tukey´s multiple comparison post-test, respectively for comparisons between groups and Bonferroni correction using Graphpad Prism 6 Software (Version 6.05). Differences were considered significant at p ≤ 0.05 and are indicated with asterisks in the figures (*p ≤ 0.05).

## Additional Information

**How to cite this article**: Wäster, P. *et al*. Extracellular vesicles are transferred from melanocytes to keratinocytes after UVA irradiation. *Sci. Rep*. **6**, 27890; doi: 10.1038/srep27890 (2016).

## Supplementary Material

Supplementary Information

Supplementary video S1

## Figures and Tables

**Table 1:** Analysis of extracellular vesicle (EV) in conditioned medium from UV-irradiated melanocytes and keratinocytes using an iZON QNano Nanopore instrument.

Cell type	Treatment	Size (mean ± SD)	Vesicle min-max (nm)	Particles/ml
Melanocytes	Control	ND	ND	ND
	UVA	233 ± 20	89–347	6.9 · 10^7^
	UVB	ND	ND	ND
Keratinocytes	Control	ND	ND	ND
	UVA	180 ± 15	96–248	5.7 · 10^5^
	UVB	ND	ND	ND

Conditioned media from 10^7^ cells was collected 2 h after irradiation, and the EVs were isolated and analyzed using nanopore NP100, 150 and 200 (detectable range 50–400 nm).

ND; not detected.

## References

[b1] Cocucci E. & Meldolesi J. Ectosomes and exosomes: shedding the confusion between extracellular vesicles. Trends Cell Biol 25, 364–372, doi: 10.1016/j.tcb.2015.01.004 (2015). 25683921

[b2] Kowal J., Tkach M. & Thery C. Biogenesis and secretion of exosomes. Curr Opin Cell Biol 29, 116–125, doi: 10.1016/j.ceb.2014.05.004 (2014). 24959705

[b3] Stein J. M. & Luzio J. P. Ectocytosis caused by sublytic autologous complement attack on human neutrophils. The sorting of endogenous plasma-membrane proteins and lipids into shed vesicles. Biochem J 274 (Pt 2), 381–386 (1991). 1848755 10.1042/bj2740381 PMC1150148

[b4] Yoon Y. J., Kim O. Y. & Gho Y. S. Extracellular vesicles as emerging intercellular communicasomes. BMB Rep 47, 531–539 (2014). 25104400 10.5483/BMBRep.2014.47.10.164 PMC4261509

[b5] Chen S. T., Geller A. C. & Tsao H. Update on the Epidemiology of Melanoma. Curr Dermatol Rep 2, 24–34, doi: 10.1007/s13671-012-0035-5 (2013). 23580930 PMC3619431

[b6] Marrot L. & Meunier J. R. Skin DNA photodamage and its biological consequences. J Am Acad Dermatol 58, S139–148, doi: 10.1016/j.jaad.2007.12.007 (2008). 18410800

[b7] Tyrrell R. M. Ultraviolet radiation and free radical damage to skin. Biochem Soc Symp 61, 47–53 (1995). 8660402 10.1042/bss0610047

[b8] Appelqvist H., Waster P., Eriksson I., Rosdahl I. & Ollinger K. Lysosomal exocytosis and caspase-8-mediated apoptosis in UVA-irradiated keratinocytes. J Cell Sci 126, 5578–5584, doi: 10.1242/jcs.130633 (2013). 24127565

[b9] Eding C. B. . Melanoma Growth and Progression After Ultraviolet A Irradiation: Impact of Lysosomal Exocytosis and Cathepsin Proteases. Acta Derm Venereol, doi: 10.2340/00015555-2064 (2015). 25669167

[b10] Waster P., Eriksson I., Vainikka L. & Ollinger K. Sunbathing: What’ve lysosomes got to do with it? Commun Integr Biol 7, e28723, doi: 10.4161/cib.28723 (2014). 25346791 PMC4201597

[b11] Appelqvist H. . Sensitivity to lysosome-dependent cell death is directly regulated by lysosomal cholesterol content. PloS One 7, e50262, doi: 10.1371/journal.pone.0050262 (2012). 23166840 PMC3500374

[b12] Settembre C., Fraldi A., Medina D. L. & Ballabio A. Signals from the lysosome: a control centre for cellular clearance and energy metabolism. Nat Rev Mol Cell Biol 14, 283–296, doi: 10.1038/nrm3565 (2013). 23609508 PMC4387238

[b13] Idone V. . Repair of injured plasma membrane by rapid Ca2+-dependent endocytosis. Journal Cell Biol 180, 905–914, doi: 10.1083/jcb.200708010 (2008). 18316410 PMC2265401

[b14] Reddy A., Caler E. V. & Andrews N. W. Plasma membrane repair is mediated by Ca(2+)-regulated exocytosis of lysosomes. Cell 106, 157–169 (2001). 11511344 10.1016/s0092-8674(01)00421-4

[b15] Xu H. & Ren D. Lysosomal physiology. Annu Rev Physiol 77, 57–80, doi: 10.1146/annurev-physiol-021014-071649 (2015). 25668017 PMC4524569

[b16] Tam C. . Exocytosis of acid sphingomyelinase by wounded cells promotes endocytosis and plasma membrane repair. J Cell Biol 189, 1027–1038, doi: 10.1083/jcb.201003053 (2010). 20530211 PMC2886342

[b17] Marks M. S. & Seabra M. C. The melanosome: membrane dynamics in black and white. Nat Rev Mol Cell Biol 2, 738–748, doi: 10.1038/35096009 (2001). 11584301

[b18] Raposo G. & Marks M. S. Melanosomes–dark organelles enlighten endosomal membrane transport. Nat Rev Mol Cell Biol 8, 786–797, doi: 10.1038/nrm2258 (2007). 17878918 PMC2786984

[b19] Raposo G., Tenza D., Murphy D. M., Berson J. F. & Marks M. S. Distinct protein sorting and localization to premelanosomes, melanosomes, and lysosomes in pigmented melanocytic cells. J Cell Biol 152, 809–824 (2001). 11266471 10.1083/jcb.152.4.809 PMC2195785

[b20] Kondo T. & Hearing V. J. Update on the regulation of mammalian melanocyte function and skin pigmentation. Expert Rev Dermatol 6, 97–108, doi: 10.1586/edm.10.70 (2011). 21572549 PMC3093193

[b21] Park H. Y., Kosmadaki M., Yaar M. & Gilchrest B. A. Cellular mechanisms regulating human melanogenesis. Cell Mol Life Sci: CMLS 66, 1493–1506, doi: 10.1007/s00018-009-8703-8 (2009). 19153661 PMC11131482

[b22] Cicero A. L. . Exosomes released by keratinocytes modulate melanocyte pigmentation. Nat Commun 6, 7506, doi: 10.1038/ncomms8506 (2015). 26103923 PMC4491833

[b23] Hirobe T. Role of keratinocyte-derived factors involved in regulating the proliferation and differentiation of mammalian epidermal melanocytes. Pigment Cell Res 18, 2–12, doi: 10.1111/j.1600-0749.2004.00198.x (2005). 15649147

[b24] Singh S. K. . The silver locus product (Silv/gp100/Pmel17) as a new tool for the analysis of melanosome transfer in human melanocyte-keratinocyte co-culture. Exp Dermatol 17, 418–426, doi: 10.1111/j.1600-0625.2008.00702.x (2008). 18331332

[b25] Tarafder A. K. . Rab11b mediates melanin transfer between donor melanocytes and acceptor keratinocytes via coupled exo/endocytosis. J Invest Dermatol 134, 1056–1066, doi: 10.1038/jid.2013.432 (2014). 24141907

[b26] Van Den Bossche K., Naeyaert J. M. & Lambert J. The quest for the mechanism of melanin transfer. Traffic 7, 769–778, doi: 10.1111/j.1600-0854.2006.00425.x (2006). 16787393

[b27] Bivik C. A., Larsson P. K., Kagedal K. M., Rosdahl I. K. & Ollinger K. M. UVA/B-induced apoptosis in human melanocytes involves translocation of cathepsins and Bcl-2 family members. J Invest Dermatol 126, 1119–1127, doi: 10.1038/sj.jid.5700124 (2006). 16528366

[b28] Jans R., Sartor M., Jadot M. & Poumay Y. Calcium entry into keratinocytes induces exocytosis of lysosomes. Arch Dermatol Res 296, 30–41, doi: 10.1007/s00403-004-0469-0 (2004). 15127211

[b29] Defour A. . Dysferlin regulates cell membrane repair by facilitating injury-triggered acid sphingomyelinase secretion. Cell Death Dis 5, e1306, doi: 10.1038/cddis.2014.272 (2014). 24967968 PMC4079937

[b30] Morris C. E. & Homann U. Cell surface area regulation and membrane tension. J Membr Biol 179, 79–102 (2001). 11220366 10.1007/s002320010040

[b31] Witwer K. W. . Standardization of sample collection, isolation and analysis methods in extracellular vesicle research. J Extracell Vesicles 2, doi: 10.3402/jev.v2i0.20360 (2013). PMC3760646 24009894

[b32] Zoller M. Tetraspanins: push and pull in suppressing and promoting metastasis. Nat Rev Cancer 9, 40–55, doi: 10.1038/nrc2543 (2009). 19078974

[b33] Cocucci E., Racchetti G., Podini P. & Meldolesi J. Enlargeosome traffic: exocytosis triggered by various signals is followed by endocytosis, membrane shedding or both. Traffic 8, 742–757, doi: 10.1111/j.1600-0854.2007.00566.x (2007). 17488290

[b34] Laulagnier K. . Role of AP1 and Gadkin in the traffic of secretory endo-lysosomes. Mol Biol Cell 22, 2068–2082, doi: 10.1091/mbc.E11-03-0193 (2011). 21525240 PMC3113771

[b35] Tian T. . Exosome uptake through clathrin-mediated endocytosis and macropinocytosis and mediating miR-21 delivery. J Biol Chem 289, 22258–22267, doi: 10.1074/jbc.M114.588046 (2014). 24951588 PMC4139237

[b36] Stinchcombe J., Bossi G. & Griffiths G. M. Linking albinism and immunity: the secrets of secretory lysosomes. Science 305, 55–59, doi: 10.1126/science.1095291 (2004). 15232098

[b37] Cardinali G. . Keratinocyte growth factor promotes melanosome transfer to keratinocytes. J Invest Dermatol 125, 1190–1199, doi: 10.1111/j.0022-202X.2005.23929.x (2005). 16354189

[b38] Glondu M. . A mutated cathepsin-D devoid of its catalytic activity stimulates the growth of cancer cells. Oncogene 20, 6920–6929, doi: 10.1038/sj.onc.1204843 (2001). 11687971

[b39] Laurent-Matha V. . Catalytically inactive human cathepsin D triggers fibroblast invasive growth. J Cell Biol 168, 489–499, doi: 10.1083/jcb.200403078 (2005). 15668295 PMC2171724

[b40] Andersson E., Vahlquist A. & Rosdahl I. Beta-carotene uptake and bioconversion to retinol differ between human melanocytes and keratinocytes. Nutr Cancer 39, 300–306, doi: 10.1207/S15327914nc392_21 (2001). 11759295

[b41] Gilchrest B. A., Vrabel M. A., Flynn E. & Szabo G. Selective cultivation of human melanocytes from newborn and adult epidermis. J Invest Dermatol 83, 370–376 (1984). 6491362 10.1111/1523-1747.ep12264638

[b42] Rosdahl I., Andersson E., Kagedal B. & Torma H. Vitamin A metabolism and mRNA expression of retinoid-binding protein and receptor genes in human epidermal melanocytes and melanoma cells. Melanoma Res 7, 267–274 (1997). 9293476 10.1097/00008390-199708000-00001

[b43] Appelqvist H. . Attenuation of the lysosomal death pathway by lysosomal cholesterol accumulation. Am J Pathol 178, 629–639, doi: 10.1016/j.ajpath.2010.10.030 (2011). 21281795 PMC3069902

