# Supplementary material for: Extracellular vesicles are transferred from melanocytes to keratinocytes after UVA irradiation
Source: Sci Rep. 2016 Jun 13;6:27890. doi: 10.1038/srep27890 (PMC4904274; doi:10.1038/srep27890)
Supplement: Supplementary Information [file srep27890-s1.pdf]

Supplementary material;

Title;

Extracellular vesicles are transferred from melanocytes to keratinocytes after UVA irradiation

Authors;

Petra Wäster[1], Ida Eriksson[1], Linda Vainikka[1], Inger Rosdahl[2], Karin Öllinger[1*]

Video legend

Video 1. UVA-irradiated melanocytes release GFP-LAMP-1-positive EVs, which are taken up by keratinocytes.

Melanocytes were transfected with CellLight BacMAM GFP-LAMP-1 and co-cultured with keratinocytes at the ratio 1:2 in 96-well plates and incubated for 24 h. After UVA irradiation, the cultures were observed using Incucyte ZOOM® software (Essen Biosciences, Michigan, USA), and time-lapse phase contrast and fluorescent images were combined. Frames were captured every 10 minutes for 2 h.

Supplementary figure legends

Supplementary Figure S1.

UVA irradiation induces plasma membrane alterations in melanocytes.

Human epidermal melanocytes in PBS with or without Ca^{2+} were exposed to UVA or UVB. **a.** Merged images of DAPI (blue, nuclei) and propidium iodide (PI, pink) staining and quantification of PI positive cells, directly following irradiation in the presence or absence of Ca^{2+}. PI only entered cells that failed to reseal their plasma membrane. **b.** Labeling of lipid rafts in cells with or without pretreatment with the aSMase inhibitor desipramine (DPA; 25 µM, 2 h), detected directly after UV irradiation. **c.** Activity of aSMase directly after UV irradiation, in cells with or without pretreatment with the aSMase inhibitor desipramine (DPA; 25 µM, 2 h), * denotes significant difference $p \leq 0.05$ (n=4).

Supplementary Figure S2.

UVA-induced increase in melanocyte size. Analysis of cell size of melanocytes after irradiation with UVA or UVB estimated as alterations in forward scatter by flow cytometry. The experiments were performed in triplicates using cells from different donors. * denotes significant difference $p \leq 0.05$ compared with the respective control.

Supplementary Figure S3.

Representative flow cytometry plots from the analysis of GFP-LAMP-1 and/or NKI/beteb positive keratinocytes. a. Negative control without staining. **b.** Co-culture of melanocytes and keratinocytes. Hoechst 33342 positive keratinocytes in gate B. **c.** Overlay of GFP-LAMP-1 positive keratinocytes, derived from gate B, analyzed 2 hours after irradiation; control (green), UVA (blue) or UVB (red). Representative histograms of GFP-LAMP-1 positive (green) and NKI/beteb positive (red) keratinocytes in **d.** Control (2 h), **e.** Control (24 h), **f**. 2 hours after UVA irradiation, **g.** 24 h after UVA irradiation, **h.** 2 hours after UVB irradiation, **i.** 24 hours after UVB irradiation.

Supplementary Figure S4.

UV induced transfer of GFP-LAMP-1 and melanosomal transfer.

Human epidermal melanocytes, transfected with BacMAM GFP-LAMP-1 and keratinocytes prestained with Hoechst 33342 were co-cultured for 24 h, irradiated with UVA or UVB and immunostained **a.** Flow cytometric quantification of NKI/beteb positive keratinocytes after 2 h. **b.** Conditioned media collected immediately after irradiation of melanocytes and added to un-irradiated melanocytes. GFP-LAMP-1-positive cells were quantified by flow cytometry after 2 h**.** Tyrosinase activity in unirradiated melanocytes after supplementation of conditioned media from UV-irradiated **c.** keratinocytes, and **d.** melanocytes. The experiments were performed in quadruplicates using cells from different donors. Human epidermal keratinocytes transfected with BacMAM GFP-LAMP-1 were irradiated with UVA or UVB; the conditioned media were immediately collected and added to cultures of **e.** un-irradiated melanocytes, or **f.** keratinocytes**,** and GFP-LAMP-1-positive cells were determined by flow cytometry. The experiments were repeated twice (e) or 3 times (f) using cells from different donors. * denotes significant difference $p \leq 0.05$.

Supplementary Figure S5.

UVA induced cathepsin D release.

Human epidermal melanocytes were irradiated with UVA or UVB. Immunoblot of cathepsin D (Cat D, 1:1000, #01-12-030104, Athens Research and Technology Inc., Athens, GA, USA) in concentrated medium collected immediately after irradiation. (Please note, inserted ladder with specified molecular weight, +; positive control, C; unirradiated control, A; UVA, B; UVB, and dashed rectangle; crop area of active 34 kDa cathepsin D). Isotype controls were analyzed in parallel, and no interference was noted. Corresponding cropped blot is presented in Figure 1d.

Supplementary Figure S6.

ASMase release after UVA irradiation.

Human epidermal melanocytes were irradiated with UVA or UVB. Immunoblot of ASMase (1:1000, #3687, Cell Signaling Technology, MA, USA) in concentrated medium collected immediately after irradiation. (Please note, inserted ladder with specified molecular weight, +; positive control, C; unirradiated control, A; UVA, B; UVB, and dashed rectangle; crop area).

Isotype controls were analyzed in parallel, and no interference was noted. Corresponding cropped blot is presented in Figure 1d.

Supplementary Figure S7.

Immunoblot of NKI/beteb.

Human epidermal melanocytes were irradiated with UVA or UVB. Immunoblot of NKI/beteb (1:1000, #MON7006-1, Monosan) in concentrated medium collected immediately after irradiation. (Please note, inserted ladder with specified molecular weight, +; positive control, C; unirradiated control, A; UVA, B; UVB, and dashed rectangle; crop area). Isotype controls were analyzed in parallel, and no interference was noted. Corresponding cropped blot is presented in Figure 1d.

Supplementary Figure S8.

Immunoblot of flotillin-1.

Human epidermal melanocytes were irradiated with UVA or UVB. Immunoblot of flotillin-1 (1:500, #610820, BD Biosciences, CA, USA) in microparticles isolated by ultracentrifugation from conditioned media of respective culture. (Please note, inserted ladder with specified molecular weight, +; positive control, C; unirradiated control, A; UVA, B; UVB, and dashed rectangle; crop area). Isotype controls were analyzed in parallel, and no interference was noted. Corresponding cropped blot is presented in Figure 2b.

Supplementary Figure S9.

Immunoblot of CD63.

Human epidermal melanocytes were irradiated with UVA or UVB. Immunoblot of CD63 (1:1000, #556019, BD Biosciences) in microparticles isolated by ultracentrifugation from conditioned media of respective culture. (Please note, inserted ladder with specified molecular weight, +; positive control, C; unirradiated control, A; UVA, B; UVB, and dashed rectangle; crop area). Isotype controls were analyzed in parallel, and no interference was noted. Corresponding cropped blot is presented in Figure 2b.

Supplementary Figure S10.

Immunoblot of LAMP-1.

Human epidermal melanocytes were irradiated with UVA or UVB. Immunoblot of LAMP-1 (1:1000, sc-8099, Santa Cruz) in microparticles isolated by ultracentrifugation from conditioned media of respective culture. (Please note, inserted ladder with specified molecular weight, +; positive control, C; unirradiated control, A; UVA, B; UVB, and dashed rectangle; crop area). Isotype controls were analyzed in parallel, and no interference was noted. Corresponding cropped blot is presented in Figure 2b.

Supplementary Figure S11.

Immunoblot of LAMP-2.

Human epidermal melanocytes were irradiated with UVA or UVB. Iimmunoblot of LAMP-2 (1:1000, #ab37024, Southern Biotech) in microparticles isolated by ultracentrifugation from conditioned media of respective culture. (Please note, inserted ladder with specified molecular weight, +; positive control, C; unirradiated control, A; UVA, B; UVB, and dashed rectangle; crop area). Isotype controls were analyzed in parallel, and no interference was noted. Corresponding cropped blot is presented in Figure 2b.

Supplementary Figure S12.

Immunoblot of cathepsin D.

Human epidermal melanocytes were irradiated with UVA or UVB. Representative immunoblot of cathepsin D (1:1000, #01-12-030104, Athens Research and Technology Inc., Athens, GA, USA) in microparticles isolated by ultracentrifugation from conditioned media of respective culture. (Please note, inserted ladder with specified molecular weight, +; positive control, C; unirradiated control, A; UVA, B; UVB, and dashed rectangle; crop area). Isotype controls were analyzed in parallel, and no interference was noted. Corresponding cropped blot is presented in Figure 2b.

Supplementary Figure S13.

Immunoblot of Bak.

Human epidermal melanocytes were irradiated with UVA or UVB. Immunoblot of Bak (1:1000, #12-01-16348, Biocarta) in microparticles isolated by ultracentrifugation from conditioned media of respective culture. (Please note, inserted ladder with specified molecular weight, +; positive control, C; unirradiated control, A; UVA, B; UVB, and dashed rectangle; crop area). Isotype controls were analyzed in parallel, and no interference was noted. Corresponding cropped blot is presented in Figure 2b.

Supplementary Figure S14.

Immunoblot of NKI/beteb.

Human epidermal melanocytes were irradiated with UVA or UVB. Immunoblot of NKI/beteb (1:1000, #MON7006-1, Monosan) in microparticles isolated by ultracentrifugation from conditioned media of respective culture. (Please note, inserted ladder with specified molecular weight, +; positive control, C; unirradiated control, A; UVA, B; UVB, and dashed rectangle; crop area). Isotype controls were analyzed in parallel, and no interference was noted. Corresponding cropped blot is presented in Figure 2b.

Supplementary Figure S15.

Immunoblot of tyrosinase.

Human epidermal melanocytes were irradiated with UVA or UVB. Immunoblot of tyrosinase (1:1000, #ab175997, Abcam) in microparticles isolated by ultracentrifugation from conditioned media of respective culture. (Please note, inserted ladder with specified molecular weight, +; positive control, C; unirradiated control, A; UVA, B; UVB, and dashed rectangle; crop area). Isotype controls were analyzed in parallel, and no interference was noted. Corresponding cropped blot is presented in Figure 2b.

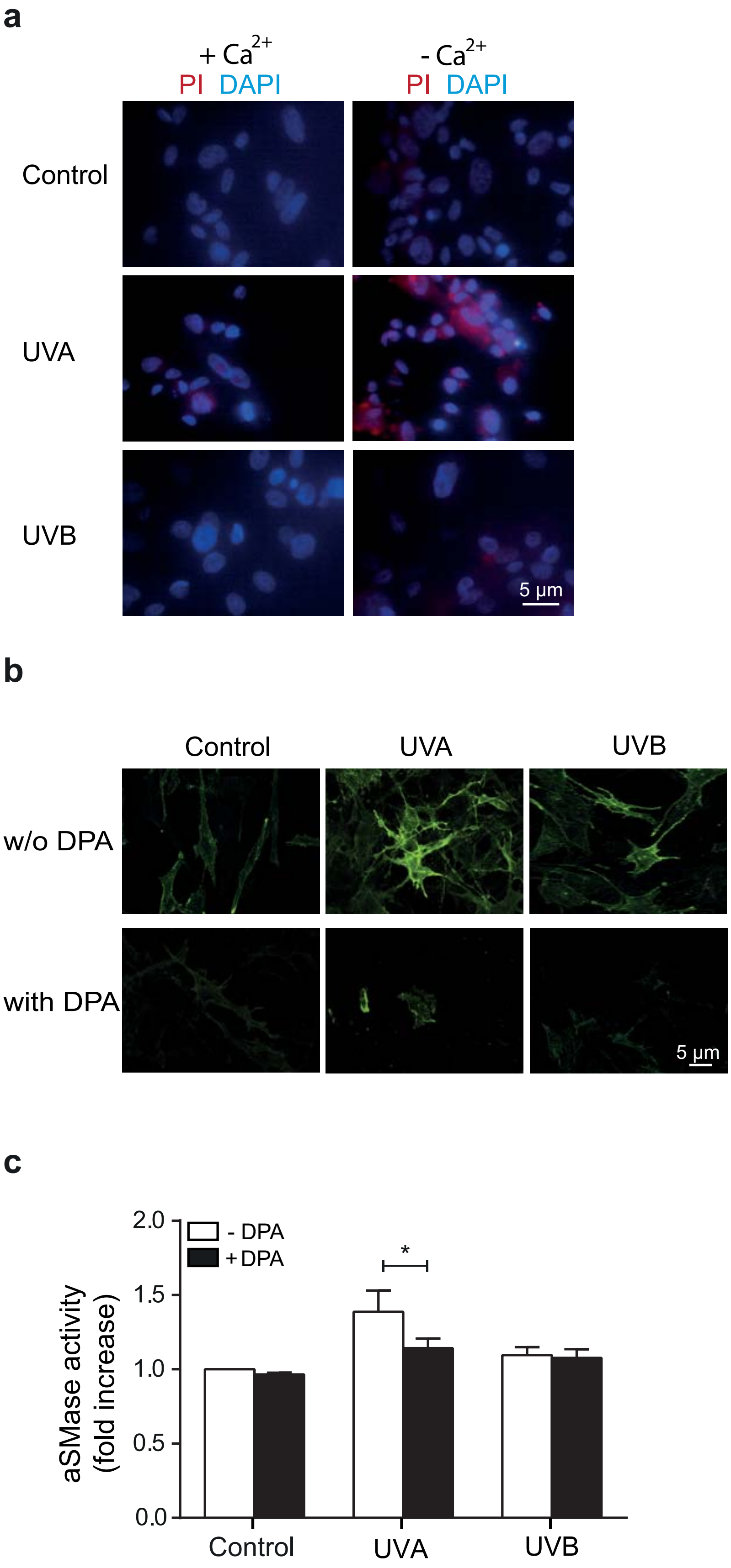

Figure S1. Wäster et al., 2016.

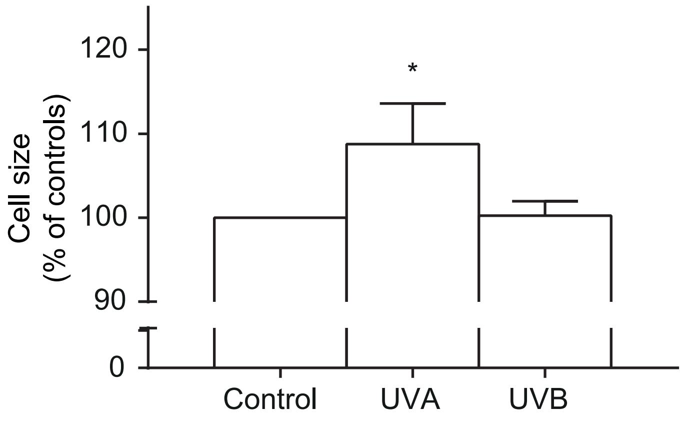

Figure S2. Wäster et al., 2016

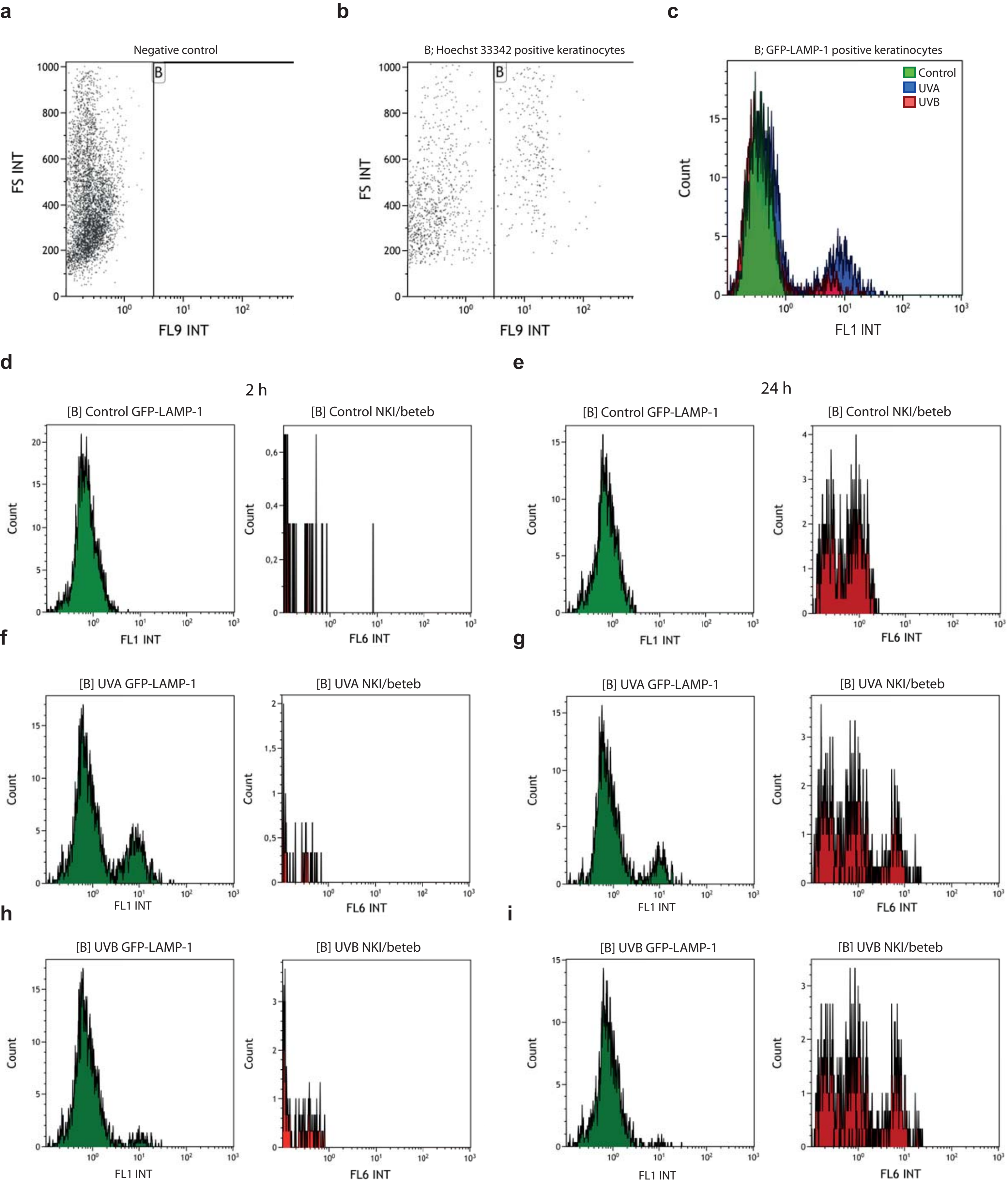

Figure S3. Wäster et al., 2016

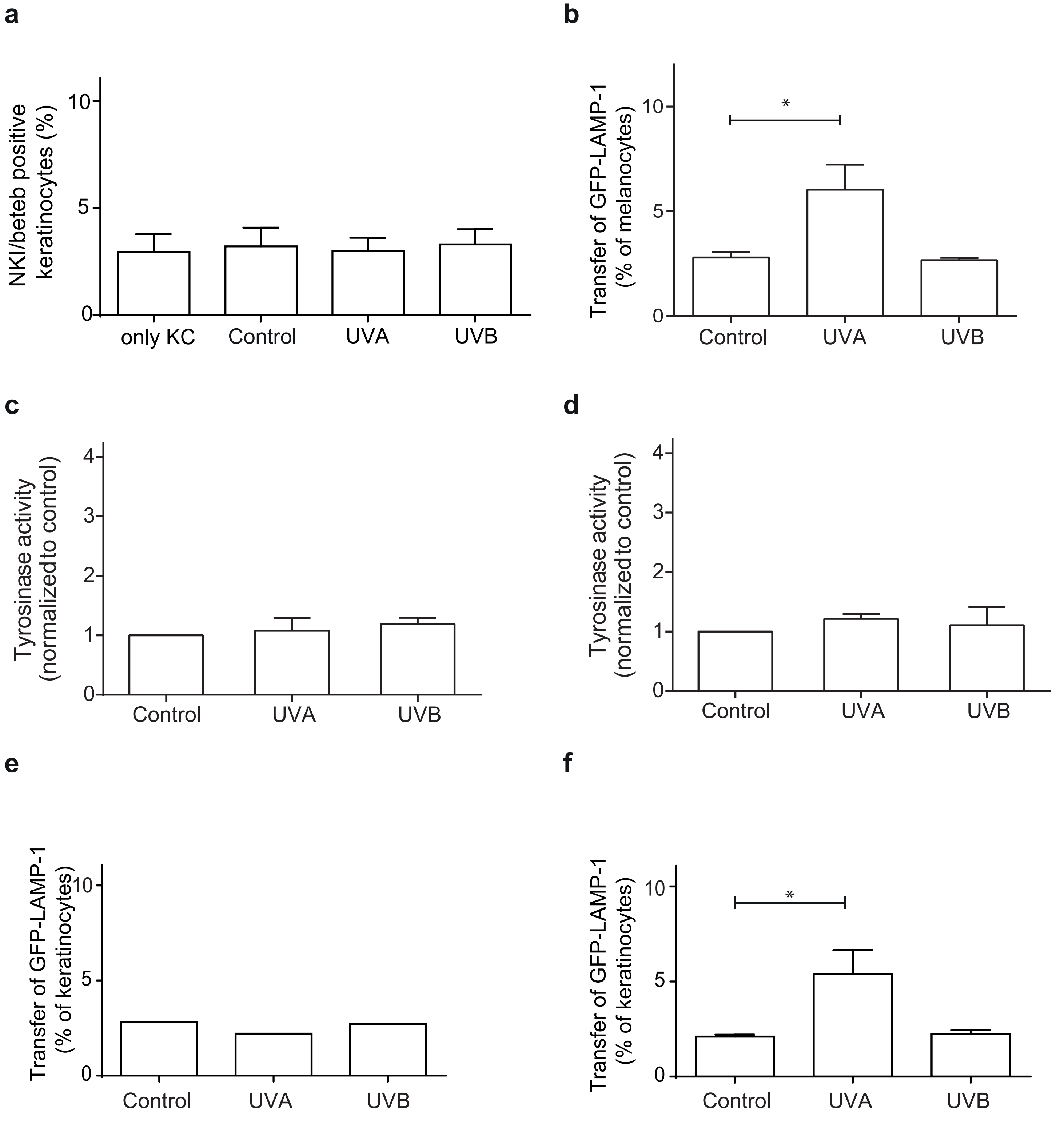

Figure S4. Wäster et al., 2016

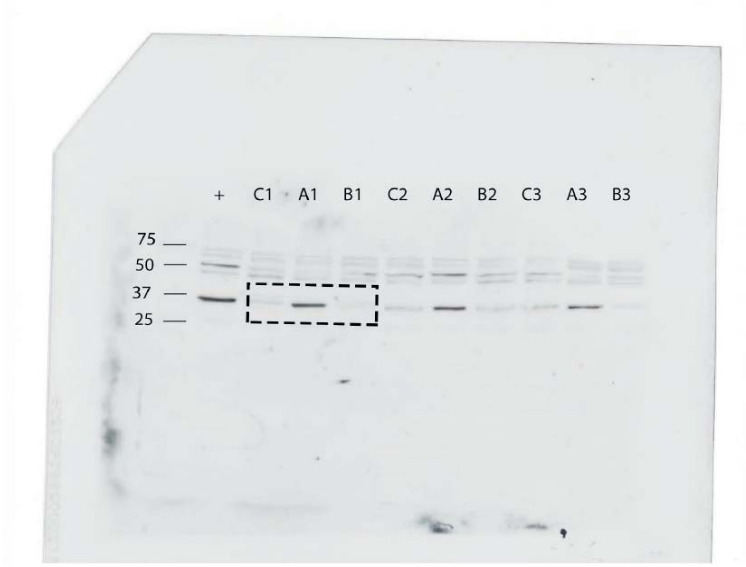

Figure S5. Wäster et al., 2016

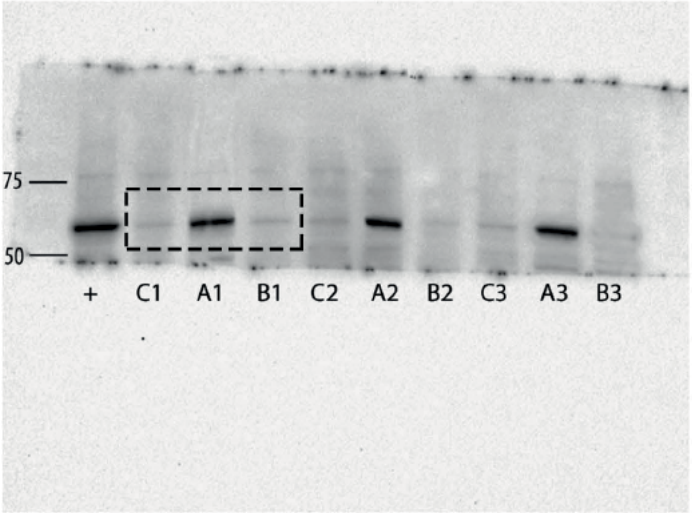

Figure S6. Wäster et al., 2016

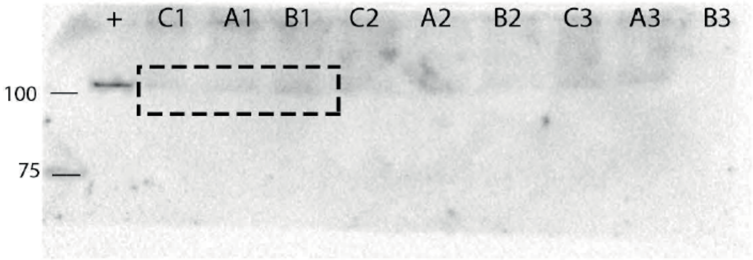

Figure S7. Wäster et al., 2016

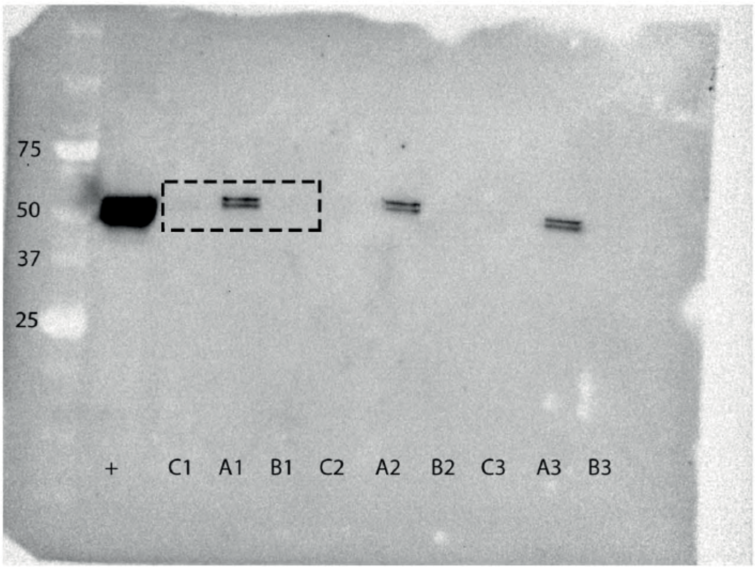

Figure S8 Wäster et al., 2016

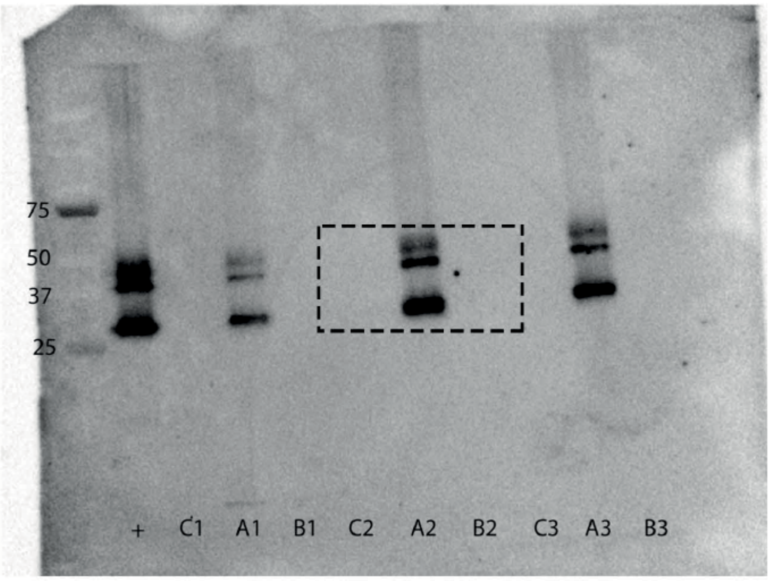

Figure S9 Wäster et al., 2016

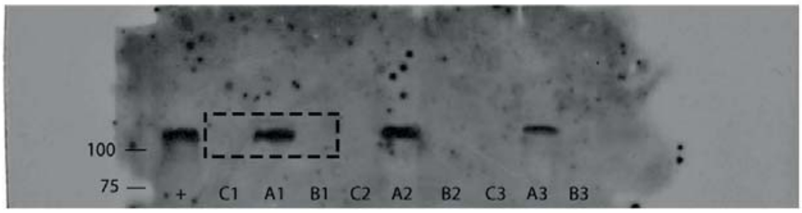

Figure S10 Wäster et al., 2016

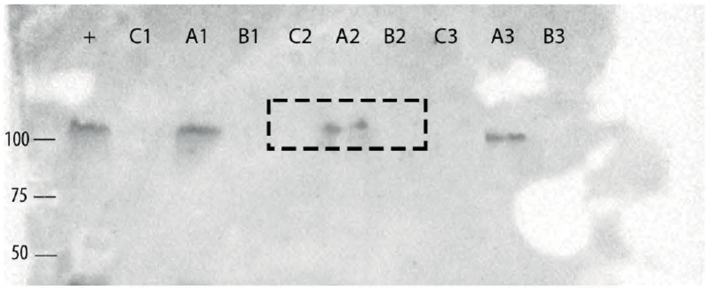

Figure S11. Wäster et al., 2016

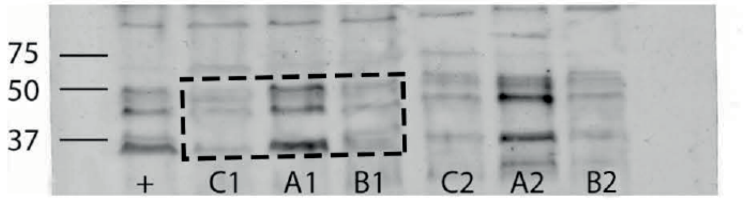

Figure S12. Wäster et al., 2016

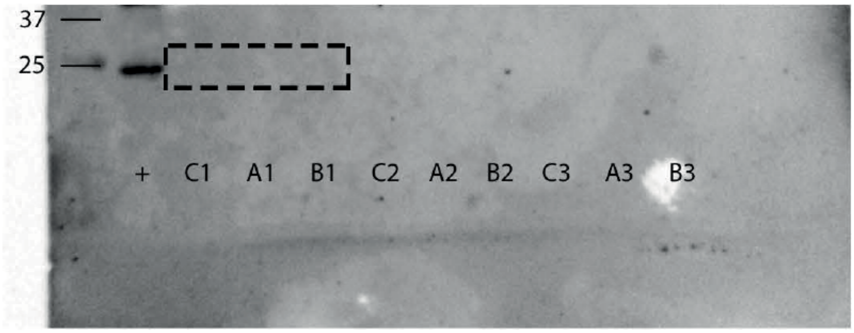

Figure S13. Wäster et al., 2016

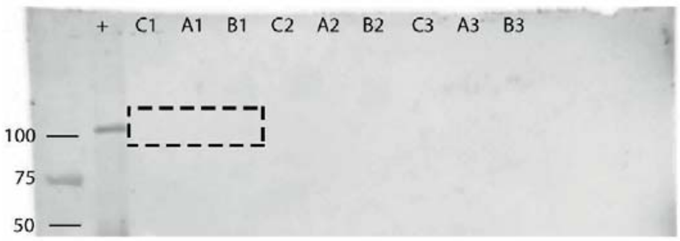

Figure S14. Wäster et al., 2016

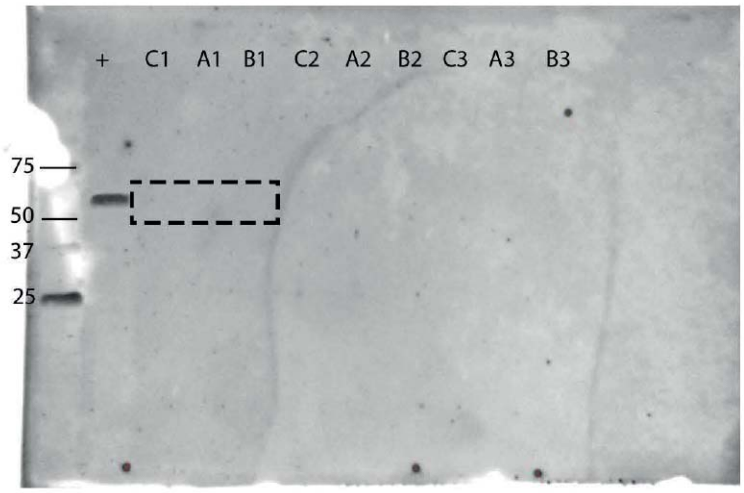

Figure S15. Wäster et al., 2016
